# TLR7 and TLR3 Sense *Brucella abortus* RNA to Induce Proinflammatory Cytokine Production but They Are Dispensable for Host Control of Infection

**DOI:** 10.3389/fimmu.2017.00028

**Published:** 2017-01-23

**Authors:** Priscila C. Campos, Marco Túlio R. Gomes, Erika S. Guimarães, Gabriela Guimarães, Sergio C. Oliveira

**Affiliations:** ^1^Departamento de Bioquímica e Imunologia, Universidade Federal de Minas Gerais, Belo Horizonte, Brazil; ^2^Instituto Nacional de Ciência e Tecnologia em Doenças Tropicais (INCT-DT), Conselho Nacional de Desenvolvimento Científico e Tecnológico, Ministério de Ciência Tecnologia e Inovação, Salvador, Brazil

**Keywords:** *B. abortus*, innate immunity, toll-like receptors, RNA, cytokines, type I IFN

## Abstract

*Brucella abortus* is a Gram-negative, facultative intracellular bacterium that causes brucellosis, a worldwide zoonotic disease leading to undulant fever in humans and abortion in cattle. The immune response against this bacterium relies on the recognition of microbial pathogen-associated molecular patterns, such as lipoproteins, lipopolysaccharides, and DNA; however, the immunostimulatory potential of *B. abortus* RNA remains to be elucidated. Here, we show that dendritic cells (DCs) produce significant amounts of IL-12, IL-6, and IP-10/CXCL10, when stimulated with purified *B. abortus* RNA. IL-12 secretion by DCs stimulated with RNA depends on TLR7 while IL-6 depends on TLR7 and partially on TLR3. Further, only TLR7 plays a role in IL-12 production induced by *B. abortus* infection. Moreover, cytokine production in DCs infected with *B. abortus* or stimulated with bacterial RNA was reduced upon pretreatment with MAPK/NF-κB inhibitors. By confocal microscopy, we demonstrated that TLR7 is colocalized with *B. abortus* in LAMP-1^+^ *Brucella*-containing vacuoles. Additionally, type I IFN expression and IP-10/CXCL10 secretion in DCs stimulated with bacterial RNA were dependent on TLR3 and TLR7. Our results suggest that TLR3 and TLR7 are not required to control *Brucella* infection *in vivo*, but they play an important role on sensing *B. abortus* RNA *in vitro*.

## Introduction

The innate immunity is important to the initial recognition of pathogens, the development of a neutralizing response, and mobilization of the adaptive immunity (1). This defense relies on a limited set of evolutionary conserved pattern recognition receptors (PRRs) that recognize specific pathogen-associated molecular patterns (PAMPs) commonly present in microbes but not in mammals. Upon detection of PAMPs, some PRRs trigger an inflammatory response leading to the efficient destruction of the invading pathogens (2). Among these PRRs, the toll-like receptors (TLRs) family has gained more attention (1, 3).

Toll-like receptors are a well-conserved family of proteins, characterized by an extracellular leucine-rich repeat domain and an intracellular toll/IL-1 receptor-like domain (4). In mammals, the TLR family comprises more than 12 members (1). Although TLR1–TLR9 are conserved between humans and mice, TLR10 is not functional in mice because of a retrovirus insertion, and TLR11, TLR12, and TLR13 are lost in the human genome (5). Surface TLRs such as TLR1, TLR2, TLR4, TLR5, TLR6, and TLR11 mainly recognize microbial membrane components including lipids, lipoproteins, and flagella. TLR3, TLR7, TLR8, and TLR9 are preferentially expressed in intracellular vesicles of the endoplasmic reticulum (ER), endosomes, and lysosomes and recognize both microbial and viral nucleic acid motifs [double-stranded RNA, single-stranded RNA (ssRNA), and CpG-DNA are recognized by TLR3, TLR7/8, and TLR9, respectively] (6). TLRs responsible for sensing nucleic acids are expressed in various cell types, including dendritic cells (DCs), macrophages, and B cells (7). Microbial nucleic acids are an important class of PAMPs and self/non-self recognition is based on different parameters, such as their sequence, structure, molecular modifications, and localization (mislocalized self nucleic acids such as extranuclear DNA or extracellular RNA are recognized as damage-associated molecular patterns) (8). There are recent reports on the properties of nucleic acid recognition for viral RNA and bacterial DNA (9, 10); however, little is known about the immunogenicity of prokaryotic RNA. Moreover, the role of TLRs acting as bacterial RNA sensors is controversial. Initially, it was reported that bacteria from group B *Streptococcus* (GBS) potently induced type I IFN in conventional DCs (cDCs) by a mechanism that required TLR7, MyD88, and the transcription factor IRF-1. These molecules colocalized with bacterial products in degradative vacuoles bearing lysosomal markers, linking lysosomal recognition of bacterial RNA with a robust IFN response (11). Further, Eberle et al. demonstrated that bacterial RNA is a potent trigger for type I IFN secretion in human peripheral blood mononuclear cells. The same study reported that murine plasmacytoid DCs from TLR7-deficient mice were unable to trigger an immune response against bacterial RNA (12). In addition, Love et al. demonstrated that the induction of IFN-α and IFN-λ1 (a type III IFN) by *Borrelia burgdorferi* RNA or live spirochetes requires TLR7-dependent signaling and an enhanced IRF7 expression (13). The TLR-mediated sensing to bacterial RNA depends on the cellular type studied and the chosen model of infection and some studies reported cases in which TLRs are not essential. For instance, Deshmukh et al. reported that the recognition of ssRNA from GBS and other Gram-positive bacteria by macrophages and monocytes depends on the adaptors MyD88 and UNC93B, but not TLRs (14). It was also demonstrated by Gratz et al. that in cDCs and macrophages stimulated with *Streptococcus pyogenes* RNA, type I IFN was induced in the absence of TLR3, TLR7, and TLR9 (15).

*Brucella abortus* is a facultative, intracellular Gram-negative bacterium that causes brucellosis, an important zoonotic infection that causes reproductive disease in domestic animals and chronic debilitating disease in humans (16, 17). *Brucella* infects and multiplies in various cell types, including macrophages, DCs, and non-phagocytic cells (18). The first line of defense against brucellosis includes phagocytosis by professional phagocytes (neutrophils, macrophages, and DCs), and natural killer (NK) cells, recognition of PAMPs by PRRs (e.g., TLRs), secretion of cytokines and chemokines, and activation of the complement system (19, 20). Signaling *B. abortus* infection *via* TLRs has been investigated by several groups including ours. These studies have highlighted the involvement of TLR2, TLR4, TLR6, and TLR9, as well as a dependence of MyD88 on immune responses against this bacterium (21–26). To evade immune surveillance, *Brucella* is devoid of conspicuous molecular determinants such as pili, fimbriae, and capsules, and it possess non-canonical surface molecules, such as lipopolysaccharide (LPS), ornithine-containing lipids, lipoproteins, and flagella, structures that lack marked PAMP activities and hence are very weak inducers of innate immunity (27). However, there are still few reports in the literature related to the host innate immune response against *Brucella*-derived nucleic acids (24, 28–30). Moreover, the role of *Brucella* RNA during bacterial infections remains undeciphered. In this study, we focused on the stimulatory activity of *B. abortus* RNA, as well as the role of TLR3 and TLR7 in cell signaling pathways and host protection against infection.

## Materials and Methods

### Bacteria

*Brucella abortus* smooth virulent strain S2308 was obtained from our laboratory collection. All work with *Brucella*, including animal experiments, was conducted in the biosafety cabinet or a primary containment device within a dedicated laboratory. Appropriate laboratory coats, gloves, and protective eyewear were provided to ensure the safety of personnel. All personnel working in the biosafety laboratory were trained and approved for entry by an individual knowledgeable in the biosafety practices. Frozen stocks were prepared with bacteria previously grown in *Brucella* broth liquid medium (BB) (Becton Dickinson, Franklin Lakes, NJ, USA) at 37°C at 180 rpm. The bacterial culture was centrifuged after 72 h of growth and the pellet was resuspended in saline solution plus 25% glycerol. Aliquots of these cultures were serially diluted, plated on BB agar 1.5%, and after incubation for 72 h at 37°C, bacterial numbers were determined by counting CFU.

### Mice

TLR3, TLR7, or MyD88 KO mice were kindly provided by Dr. Shizuo Akira, Osaka University, Japan. Wild-type (WT) strain C57BL/6 mice were obtained from the Federal University of Minas Gerais (UFMG, Belo Horizonte, Brazil). Genetically deficient and control mice were maintained at our facilities and used at 6–8 weeks of age. All animal experiments were preapproved by the Institutional Animal Care and Use Committee of the UFMG (CETEA no. 104/2011). Mice were housed in filter-top cages and provided with sterile water and food *ad libitum*.

### Generation of Bone Marrow-Derived DCs

Bone marrow cells were obtained from femur and tibiae of KO and WT mice, and they were differentiated into DCs as previously described (26, 31, 32). Briefly, bone marrow cells were cultured in RPMI medium 1640 (Thermo Fisher Scientific, Waltham, MA, USA) containing 10% fetal bovine serum (FBS), 100 U/mL penicillin, 100 µg/mL streptomycin, and 20 ng/mL murine recombinant granulocyte-macrophage colony-stimulating factor (GM-CSF). Petri dishes containing 1 × 10^7^ cells were incubated at 37°C in a 5% CO_2_ atmosphere. On day 3 of incubation, a further 5 mL of fresh complete medium containing GM-CSF was added, and on days 5 and 7, the medium was replaced with fresh supplemented medium containing GM-CSF. On day 10, non-adherent cells (immature DCs) were harvested, seeded in 24-well plates (5 × 10^5^ cells/well), and then incubated at 37°C in a 5% CO_2_ atmosphere until use.

### Isolation of *B. abortus* Total RNA from Bacterial Cell Culture

Bacteria were cultured for 3 days at 37°C in 10 mL BB liquid medium, and the bacterial suspension was pelleted into 10 eppendorfs. The supernatants were removed and each pellet was resuspended in 1 mL TRIzol^®^ (Thermo Fisher Scientific, Waltham, MA, USA). The procedure of RNA isolation was performed following instructions of the manufacturer. Briefly, each TRIzol-bacterial suspension was vortexed, then 0.2 mL chloroform was added and the mixture was centrifuged at 11,000 × *g* during 10 min at 4°C. The aqueous phase was transferred to another eppendorf and 0.5 mL ice-cold isopropanol was added. The mixture was incubated at room temperature for 10 min and then centrifuged at 11,000 × *g* during 15 min at 4°C. The supernatant was discarded and the precipitate was washed with 1 mL ice-cold ethanol 75%, through centrifugation at 7,500 × *g* during 10 min at 4°C. The pellet was air-dried and resuspended using 50 µL nuclease-free water. Total RNA was quantified using the Nanodrop 2000 spectrophotometer (Nanodrop 2000; Thermo Scientific), treated with DNase I and/or RNases A/T_1_ (Thermo Scientific) and stored at −80°C until use.

### *In Vitro* Stimulation of DCs

Stimulation of the DCs was performed by adding RPMI plus 10% FBS (0.5 mL/well) containing *B. abortus* [multiplicity of infection (MOI) 100:1], *Escherichia coli* LPS (1 µg/mL; Sigma-Aldrich), or *B. abortus* total RNA (2 µg/mL) complexed with DOTAP Liposomal Transfection Reagent (Sigma-Aldrich, St. Louis, MO, USA). Briefly, DOTAP was mixed with bacterial RNA (5:1 ratio) in 100 µL/well serum-free RPMI and incubated for 20 min at room temperature. Then, complexes were added to the cells in the presence of 12.5% FBS and cell cultures were incubated for 24 h at 37°C in a 5% CO_2_ atmosphere. Culture supernatants were collected after 24 h of stimulation and kept at −80°C until use. To the remaining cells, it was added 0.350 mL/well of TRIzol^®^ to further RNA isolation.

### RNA Isolation from DCs and Quantitative Real-time PCR

Reverse transcription of 1 µg from total RNA was performed using Illustra™ Ready-To-Go RT-PCR Beads (GE Healthcare, Little Chalfont, UK), following instructions of the manufacturer. Quantitative real-time PCR was conducted in a final volume of 10 µL containing the following: SYBR^®^ Green PCR Master Mix (Thermo Fisher Scientific, Waltham, MA, USA), oligo-dT cDNA as the PCR template, and 5 µM of primers. The PCR reaction was performed with StepOne Plus Real-Time PCR System (Thermo Fisher Scientific, Waltham, MA, USA), using the following cycling parameters: 60°C for 10 min, 95°C for 10 min, 40 cycles of 95°C for 15 s and 60°C for 1 min, and a dissociation stage of 95°C for 15 s, 60°C for 1 min, 95°C for 15 s, and 60°C for 15 s. Primers were used to amplify a specific 100–120 bp fragment corresponding to specific gene targets as follows: IFN-β, forward (5′-AGCTCCAAGAAAGGACGAACAT-3′); IFN-β, reverse (5′-GCCCTGTAGGTGAGGTTGATCT-3′); β-actin, forward (5′-AGGTGTGCACCTTTTATTGGTCTCAA-3′); β-actin, reverse (5′-TGTATGAAGGTTTGGTCTCCCT-3′). All data are presented as relative expression units after normalization to the β*-actin* gene. PCR measurements were conducted in triplicate.

### Inhibition of MAPK/NF-κB Signaling Pathways in DCs

DCs were pretreated with vehicle (0.1% DMSO) or selective inhibitors for ERK1/2 (U0126; 10, 50, and 100 µM), JNK (SP600125; 1, 5, and 10 µM), p38 (SB203580; 5, 25, and 50 µM), and NF-κB (BAY1170-82; 10, 100, and 500 µM) (all from Cell Signaling Technology, Danvers, MA, USA) for 45 min. Subsequently, cells were stimulated with *B. abortus* S2308 strain (MOI 100:1) or with bacterial RNA (2 µg/mL) for 24 h, and harvested supernatants were used to measure cytokine production by ELISA.

### Measurement of IL-12, IL-6, IP-10/CXCL10, and TNF-α by ELISA

Collected supernatants of stimulated DCs were frozen until they were assayed for determination of cytokine concentrations using Mouse DuoSet ELISA (R&D Systems), according to the manufacturer’s specifications.

### Confocal Microscopy

Dendritic cells were differentiated on 12 mm glass coverslips in 24-well plates, as described before. Infection with the *B. abortus* was performed at a MOI of 100:1. After 24 h of infection, cells were washed twice with PBS and fixed in 4% paraformaldehyde, pH 7.4, at 37°C for 15 min. Once fixed, cells were incubated with the primary antibody rat anti-lysosomal-associated membrane protein 1 (LAMP-1) diluted 1:200 (Abcam, Cambridge, UK) and rabbit anti-TLR7 diluted 1:100 (Abcam, Cambridge, UK) in PBS 0.1% saponin overnight. Then, cells were washed three times with PBS 0.1% saponin and were incubated for 1.5 h at room temperature with an anti-rabbit secondary antibody conjugated to Alexa 546 (Invitrogen, Carlsbad, CA, USA) and with an anti-rat secondary antibody conjugated to Alexa 488 (Abcam, Cambridge, UK), both diluted 1:1,000. The cells were washed twice with PBS 0.1% saponin and once with PBS then mounted with the mounting medium ProLong Gold with DAPI (Invitrogen, Carlsbad, CA, USA). Stained cells were examined by confocal microscopy (Nikon C2 confocal microscope) and image analysis was performed using ImageJ software version 1.47n.

### Enumeration of *B. abortus* from Spleens

To count residual *B. abortus* CFU in the spleens of C57BL/6 WT, TLR3 KO, TLR7 KO, and MyD88 KO mice, five mice from each group were examined at 1 and 3 weeks postinfection. Spleens were homogenized with 10 mL of saline solution. To enumerate viable bacteria, homogenized spleen samples were serially diluted 10-fold with saline solution and plated on BB agar (Becton Dickinson, Franklin Lakes, NJ, USA). CFUs were counted after 3 days of incubation at 37°C.

### Statistical Analysis

All experiments were repeated at least three times with similar results and figures show data from one representative experiment. Graphs and data analysis were done using GraphPad Prism 5 (GraphPad Software), using one-way ANOVA, two-way ANOVA (Bonferroni *post hoc* test), or Student’s *t*-test (Tukey’s *post hoc* test).

## Results

### Lack of TLR7 Affects Proinflammatory Cytokine Production by DCs Activated with *B. abortus* or Its RNA

**Figure 1:**
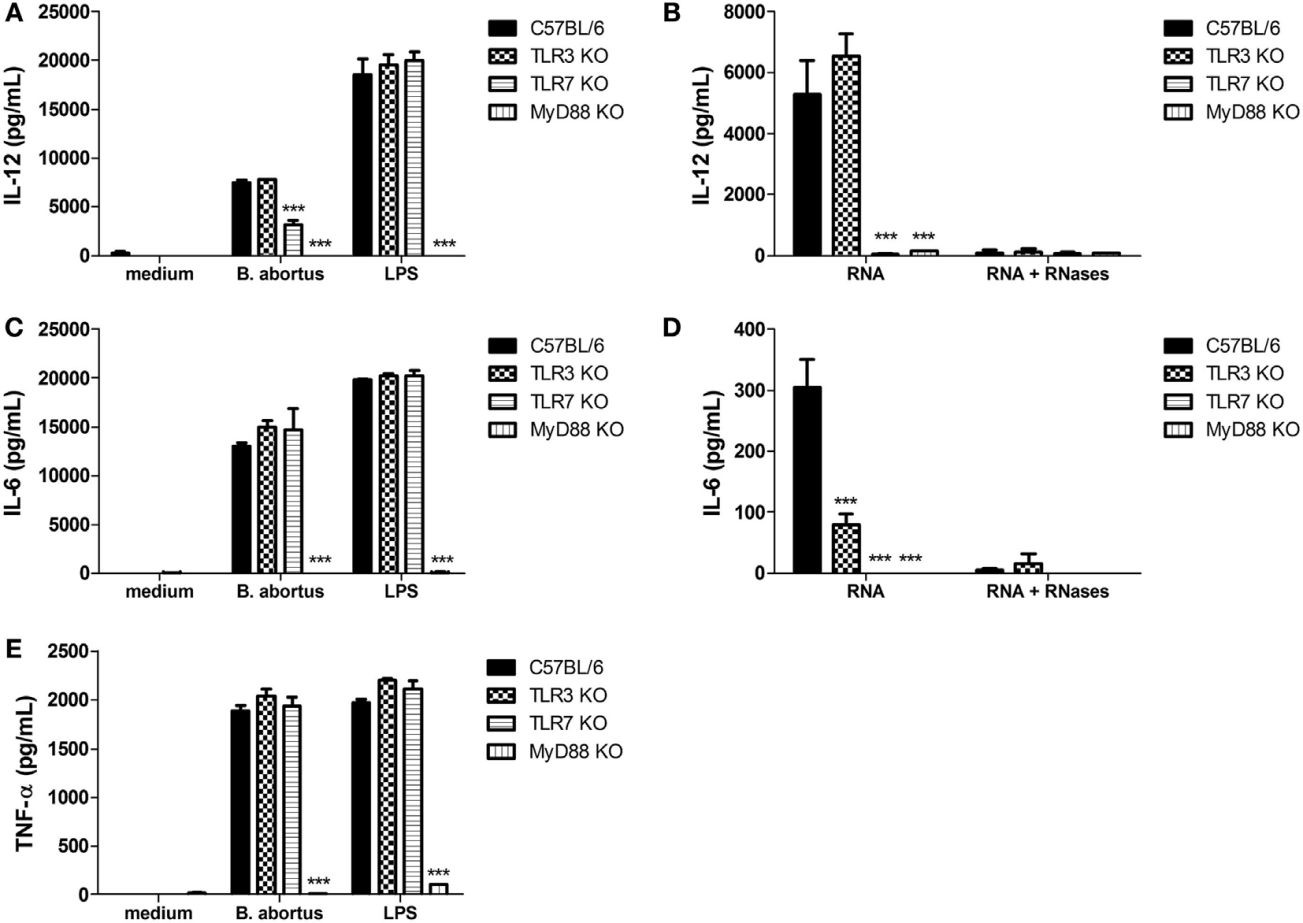
**Secretion of proinflammatory cytokines in dendritic cells (DCs) after stimulation with *Brucella abortus* or bacterial RNA**. DCs were stimulated with *B. abortus* strain S2308 (MOI 100:1) or lipopolysaccharide (LPS) (1 µg/mL) **(A,C,E)**, or bacterial RNA (2 μg/mL) **(B,D)**. Supernatants were harvested after 24 h after stimulation and cytokine secretion was determined by ELISA. Significant differences in relation to wild-type mice are denoted (for ***p < 0.001, two-way ANOVA test).

To determine whether TLR3 and TLR7 are required to induce proinflammatory cytokine production in DCs stimulated with *B. abortus*, cells from C57BL/6, TLR3 KO, TLR7 KO, and MyD88 KO were cultured with *B. abortus* (MOI 100:1) and after 24 h the supernatants were tested for production of IL-12, IL-6, and TNF-α by ELISA. IL-12 was partially reduced in TLR7 KO cells but not in TLR3 KO DCs (Figure 1A). Additionally, secretion of IL-6 and TNF-α was unaffected by the absence of TLR3 and TLR7 (Figures 1C,E). Furthermore, IL-12, IL-6, and TNF-α production were totally abrogated in MyD88 KO cells.

Next, we assessed the potential of *B. abortus* RNA as TLR3 and/or TLR7 agonist to induce proinflammatory cytokines. DCs from C57BL/6, TLR3 KO, TLR7 KO, and MyD88 KO were stimulated with purified *B. abortus* RNA and after 24 h the supernatants were tested for production of IL-12, IL-6, and TNF-α. *B. abortus* RNA was able to induce the production of IL-12 and IL-6 in WT cells (Figures 1B,D) but not TNF-α (data not shown). Further, IL-12 and IL-6 secretion were completely abrogated in TLR7 and MyD88 KO cells when compared to the WT DCs. However, IL-6 secretion was only partially dependent on TLR3 KO cells (Figure 1D). These results suggest that, upon *B. abortus* infection, only the lack of TLR7 partially affected IL-12 production in DCs. On the other hand, TLR3 and TLR7 are required to induce a cytokine response in DCs stimulated with *Brucella*-purified RNA. Additionally, our results also indicated that bacterial RNA treatment with RNases A/T1 abolished IL-12 and IL-6 in DCs, indicating that no other possible contaminant would be responsible for the cytokine production observed (Figures 1B,D).

### ERK1/2, JNK, and NF-κB but Not p38 Signaling Are Involved in Cytokine Production in DCs Stimulated with *B. abortus* or Bacterial RNA

**Figure 2:**
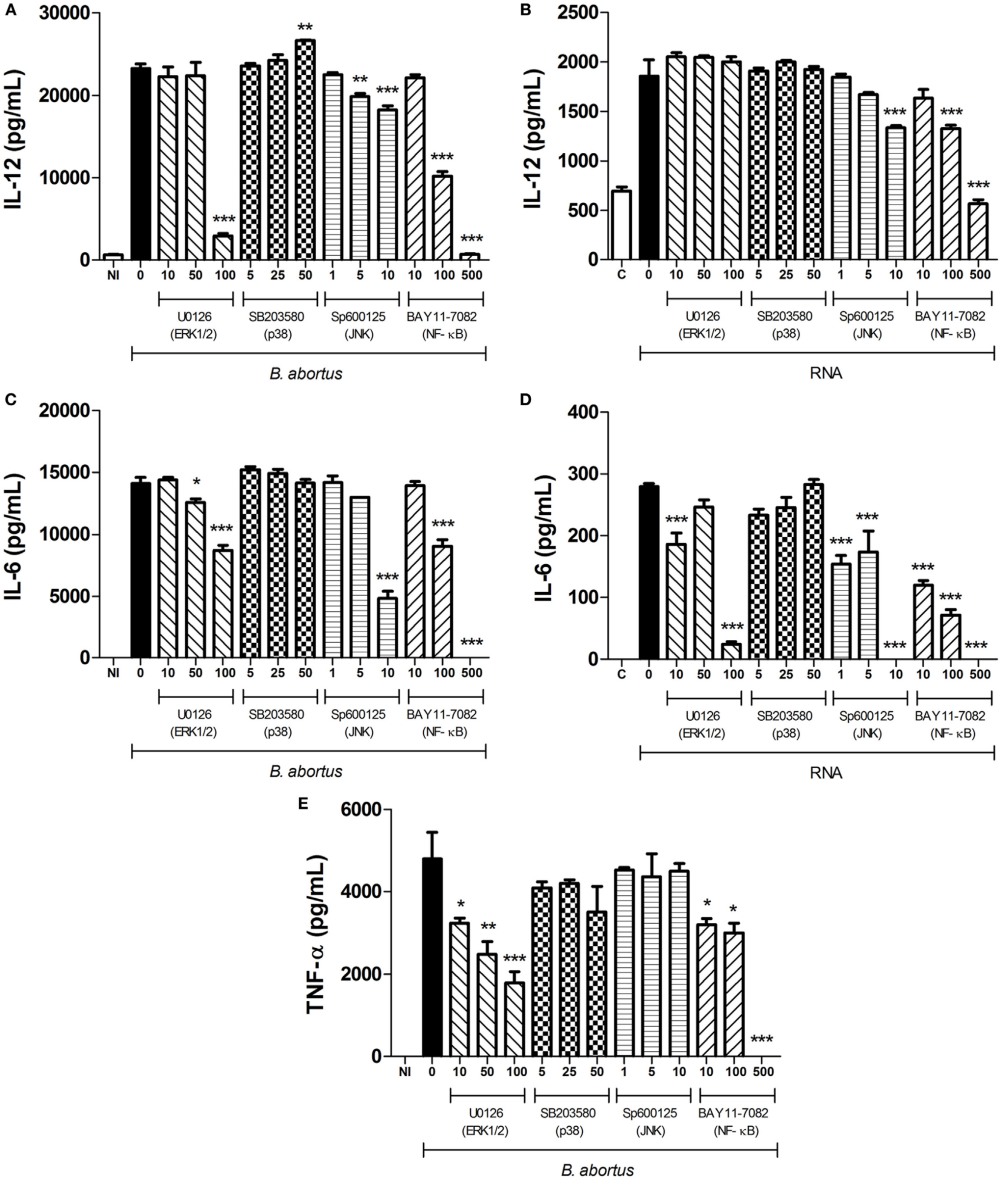
**MAPK/NF-κB signaling pathways involved on cytokine production in dendritic cells (DCs) after stimulation with *Brucella abortus* or bacterial RNA**. DCs from C57BL/6 mice were pretreated for 45 min with increasing concentrations (in micromolars) of U0126, SB203580, SP600125, and BAY11-7082 (ERK1/2, p38, JNK, and NF-κB inhibitors, respectively) or vehicle, as indicated, and then stimulated with *B. abortus* (MOI 100:1) **(A,C,E)** or bacterial RNA (2 µg/mL) **(B,D)**. Supernatants were harvested after 24 h after stimulation and cytokine secretion was determined by ELISA. Abbreviations: NI, non-infected; C, Control, DOTAP only;, RNA, DOTAP + RNA. Significant differences in relation to vehicle treatment are denoted (for *p < 0.05, **p < 0.01, and ***p < 0.001, two-way ANOVA test).

Since *B. abortus* or its purified RNA induce the production of proinflammatory cytokines in DCs, we evaluated the role of MAPK components and the NF-κB transcription factor during cell activation. DCs from WT mice were treated with selective inhibitors against ERK1/2 (U0126), p38 (SB203580), JNK (SP600125), and NF-κB (BAY1170-82) before stimulation with *B. abortus* or bacterial RNA. Harvested supernatants were used to assess IL-12, IL-6, and TNF-α production (Figure 2). Treatment of DCs with the ERK1/2 inhibitor before infection with *B. abortus* reduced the production of all three cytokines, whereas the cytokine production was not significantly altered by inhibition of p38. Moreover, inhibition of JNK in DCs stimulated with *B. abortus* resulted in a decrease in IL-12 and IL-6 but not TNF-α. It was also observed that IL-12, IL-6, and TNF-α production are reduced in infected cells treated with a selective inhibitor of NF-κB (Figures 2A,C,E).

We also assessed whether the pretreatment with MAPK/NF-κB inhibitors would affect cytokine production in DCs stimulated with *B. abortus* RNA. Both ERK1/2 and JNK inhibitors, as well as the NF-κB inhibitor, were able to reduce IL-6 production in DCs stimulated with bacterial RNA (Figure 2D). As observed for live bacteria, p38 inhibitor did not reduce the production of any studied cytokine in the supernatant of these cells. Besides, IL-12 production was diminished only in supernatants from DCs pretreated with JNK and NF-κB inhibitors (Figure 2B), which suggests that DCs infected with live bacteria may be exposed to other bacterial agonists that are recognized *via* ERK besides to JNK and NF-κB. Importantly, the reduction in cytokine production mediated by U0126, SP600125, and BAY1170-82 is not a consequence of toxicity caused by the inhibitors since treatment induces slight levels of cell death and only in high concentrations (Figure S1 in Supplementary Material). Once again, TNF-α production was not detected in DCs stimulated with *B. abortus* RNA, pretreated or not with the inhibitors previously mentioned (data not shown). All together, these results indicate that MAPK and NF-κB activation participates in proinflammatory cytokine production in DCs stimulated with *B. abortus* or its RNA.

### TLR7 and LAMP-1 Are Colocalized in *B. abortus*-Infected DCs

**Figure 3:**
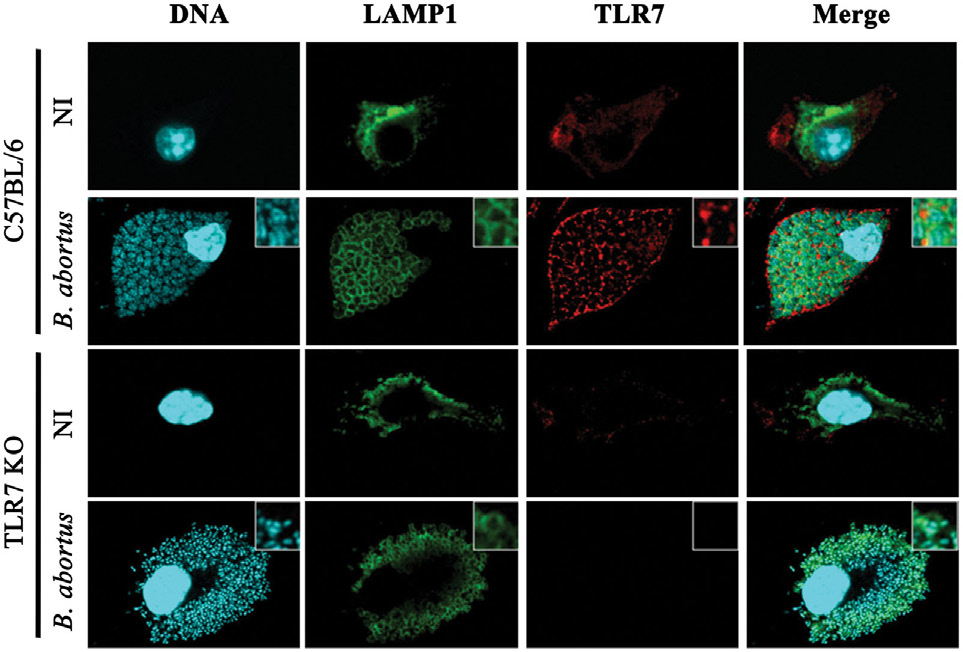
**Dendritic cells (DCs) activated by *Brucella abortus* promote TLR7 colocalization with lysosomal-associated membrane protein 1 (LAMP-1)-positive compartments**. DCs were stimulated with *B. abortus* (MOI 100:1) for 24 h. Subsequently, macrophages were fixed and stained to detect host cell and bacterial DNA (blue), TLR7 (red), and LAMP-1 (green) by confocal microscopy. The colocalization between TLR7 and LAMP-1 is highlighted in inserts. NI, non-infected.

Results shown in Figure 1 suggest that IL-12 production in DCs stimulated with *B. abortus* or its purified RNA depends on the signaling *via* TLR7. Upon entry into mammalian cells, *B. abortus* resides within a membrane-bound, ER-derived compartment, the *Brucella*-containing vacuole (BCV). Before reaching the ER, the bacteria ensure its intracellular survival by inhibiting fusion of the intermediate BCV with late endosomes and lysosomes, in which we observe the presence of LAMP-1 (33). Therefore, to gain insight into the role of TLR7 in the response to *B. abortus* infection, we performed confocal microscopy in infected DCs, labeling cells with anti-TLR7 and anti-LAMP-1 antibody, and visualizing by confocal microscopy. Our results suggest that upon infection of WT DCs with *B. abortus*, TLR7 traffics to LAMP-1^+^ endosomes, spreading throughout the cells (Figure 3). Indeed, colocalization of TLR7 and *Brucella* in LAMP-1^+^ compartments could facilitate sensing of RNA released from bacteria and subsequent cytokine production. As expected, there was no TLR7 staining in TLR7 KO DCs. Further, TLR7 KO DCs showed phenotypically distinct clustered, membrane-bound LAMP-1^+^ BCV-like structures, when compared to WT DCs. All together, these results suggest that TLR7 is involved in a *B. abortus*-induced signaling pathway, leading to a proinflammatory cytokine production.

### TLR3 and TLR7 Are Required for Type I IFN Expression and IP-10/CXCL10 Secretion in DCs Stimulated with *B. abortus* RNA

**Figure 4:**
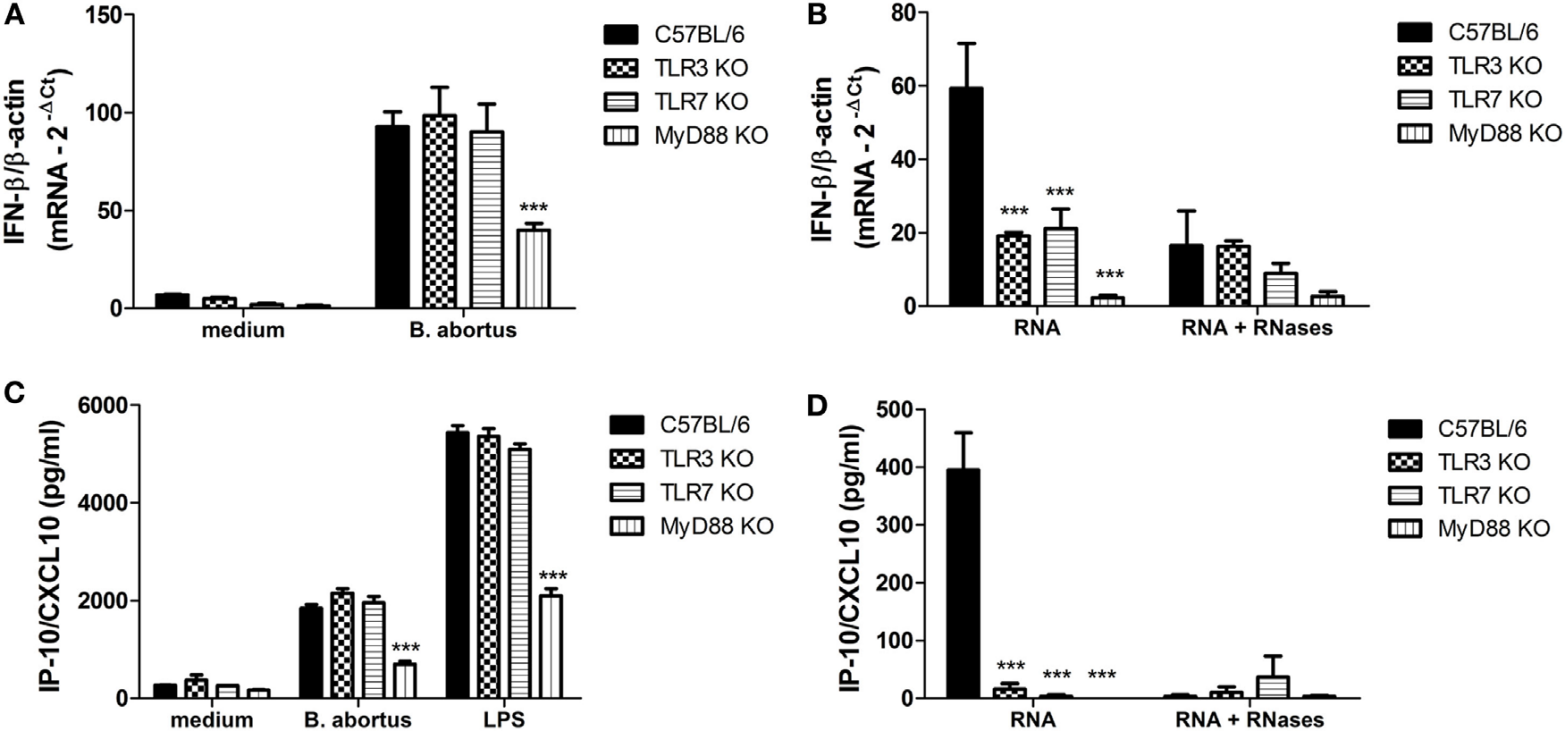
**Type I IFN expression and IP-10/CXCL10 secretion in dendritic cells (DCs) stimulated with *Brucella abortus* or bacterial RNA**. DCs were stimulated with *B. abortus* strain S2308 (MOI 100:1) **(A,C)** or bacterial RNA (2 µg/mL) **(B,D)** for 24 h. Total RNA was isolated from these cells, first-strand cDNA synthesis was performed as indicated in Section “Materials and Methods” and subjected to qPCR analysis. Supernatants were analyzed by ELISA. Significant differences related to wild-type mice are denoted (***p < 0.001, two-way ANOVA test).

It is known that upon TLR3 and TLR7 recognition of nucleic acids released into endosomes after virus or bacteria internalization or lysis results in type I IFN production, particularly IFN-β (34, 35). Therefore, we decided to investigate the levels of *IFN-*β expression and IP-10/CXCL10 production, a surrogate marker of type I IFN (36, 37), in TLR3 and TLR7 KO DCs activated with *B. abortus* or its purified RNA. We have observed no significant reduction on *IFN-*β expression and IP-10/CXCL10 production in TLR3 and TLR7 KO cells infected with *B. abortus* when compared to the WT (Figures 4A,C). Bacterial RNA was able to induce the expression of *IFN-*β and IP-10/CXCL10 production in WT DCs; however, the levels of these cytokines were diminished in TLR3 KO and TLR7 KO cells (Figures 4B,D). *IFN-*β expression and IP-10/CXCL10 production were only partially reduced in MyD88 KO cells because the IFN-responsive gene expression can also be activated *via* TLR3–TRIF pathway (38, 39). However, when MyD88 KO cells were activated with bacterial RNA a dramatic reduction on cytokine levels were observed. The previous treatment of RNA with RNases abolished the differences between KO and WT cells.

### TLR3 and TLR7 Are Not Required to Control *B. abortus* Infection *In Vivo*

**Figure 5:**
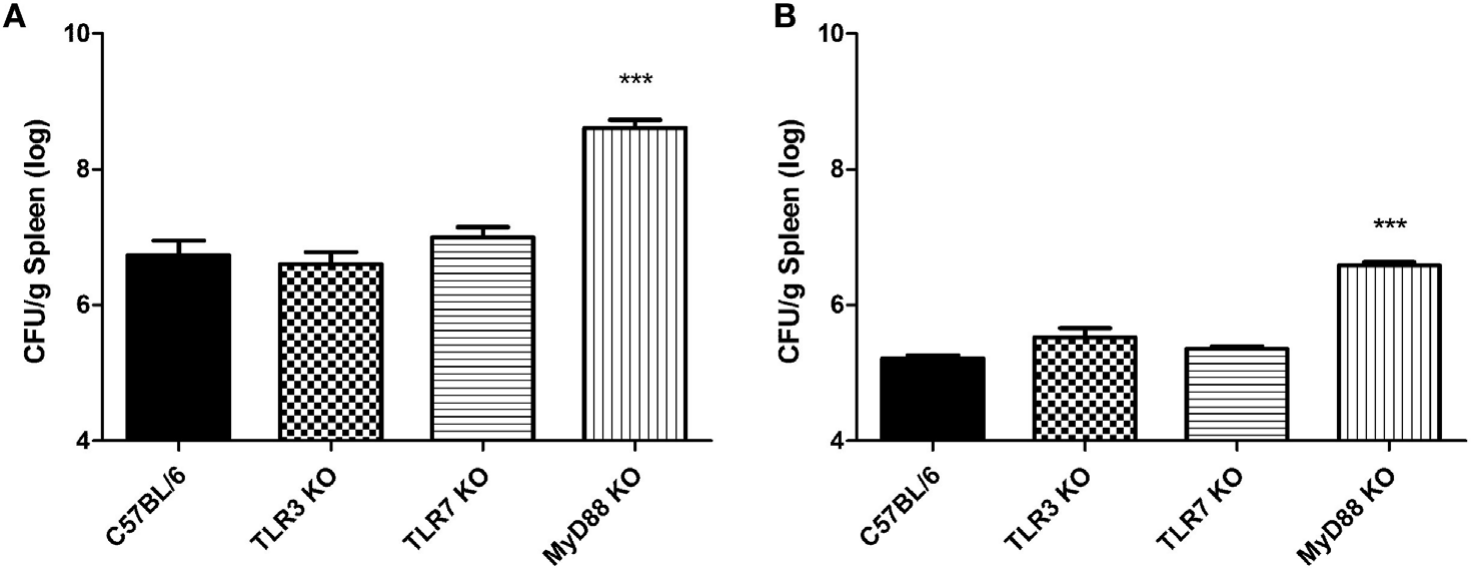
**The control of *Brucella abortus* infection *in vivo* in mice deficient for TLR3, TLR7, and for the adaptor molecule MyD88**. Residual *B. abortus* CFU in the spleen of wild-type (WT) and KO mice were determined after 1 **(A)** and 3 **(B)** weeks postinfection. Significant differences in relation to WT mice are denoted (***p < 0.001, Student’s *t*-test).

Results obtained from *in vitro* studies indicate that TLR3 and TLR7 play a role in cytokine production and type I IFN expression upon stimulation with *B. abortus* RNA but are dispensable for the induction of an immune response against live bacteria. Therefore, to test the role of these receptors in host defense against *Brucella* infection *in vivo*, C57BL/6 WT, TLR3 KO, TLR7 KO, and MyD88 KO mice to test the role of these receptors in host defense against *Brucella* infection *in vivo*. Mice were infected intraperitoneally with 10^6^ CFU *B. abortus* and the number of bacteria in the spleen was quantified. TLR3 and TLR7 KO mice were able to effectively control *Brucella* infection as the WT animals at 1 and 3 weeks postinfection (Figure 5). MyD88 KO were used as a control group and showed the highest CFU numbers in the spleen compared to other strains tested here, indicating their susceptibility to infection.

## Discussion

Here, we provided evidence that *B. abortus* RNA function as an immunostimulatory PAMP that induces the production of proinflammatory cytokines in bone marrow-derived DCs. DCs stimulated with bacterial RNA produced significant amounts of IL-12 and IL-6 (Figures 1B,D), and this production is decreased when cells are pretreated with MAPK/NF-κB inhibitors (Figures 2B,D). Moreover, *B. abortus* RNA induces IFN-β expression (Figure 4B) and IP-10/CXCL10 production (Figure 4D). On the other hand, although we observed a robust secretion of TNF-α in DCs stimulated with *B. abortus* (Figure 1E), there was no detectable TNF-α production in RNA-stimulated cells (data not shown). Results reported by Eberle et al. indicated that TNF-α secretion in murine DCs induced by bacterial RNA is independent of endosomal maturation and consequently independent of endosomal nucleic acid-sensing TLRs (12). Furthermore, the transfection of DCs with RNA complexed to DOTAP, a cationic lipid that mimics the chemical and physical attributes of biological lipids (40), allows direct delivery of RNA to endosomes, avoiding a possible nucleic acid sensing by cytosolic sensors, for example, members of retinoic acid-inducible gene I (RIG-I)-like receptors family: RIG-I, melanoma differentiation factor 5 (MDA5), and laboratory of genetics and physiology 2 (LGP2) (38).

Our data reported here also reveal that *B. abortus* RNA is recognized in a TLR3- and TLR7-dependent manner by DCs. However, the involvement of these two receptors is only noticed upon stimulation with purified RNA complexed to DOTAP and not upon infection with live bacteria (Figures 1 and 4). Alternatively, the immune responses against *B. abortus* infection could be triggered by major agonists such as DNA, lipoproteins, or LPS, and probably such stimuli are more efficient in activating proinflammatory cytokine secretion and type I IFN expression than bacterial RNA. A recent review published by our group describes *B. abortus* DNA as a major agonist to activate innate responses against this bacterium, and this activation is mediated by the endosomal TLR9 as well as cytosolic molecules such as AIM2 and STING (30). Moreover, endosomal TLRs may interact with each other, resulting in a unidirectional and hierarchical inhibition. In a work performed by Wang and colleagues in 2006, it was demonstrated a cooperative interaction among TLR7, TLR8, and TLR9, in HEK293 cells transfected with human or murine TLRs in a paired combination (39). In this study, the results indicated that TLR8 inhibits TLR7 and TLR9, and TLR9 inhibits TLR7 but TLR7 does not inhibit TLR8 neither TLR9. Furthermore, in mice, it is possible that the inflammatory response triggered upon TLR7 activation may be modulated by TLR8 in all DC subsets. We cannot rule out the possibility that *B. abortus* RNA is sensed by other PRRs in a MyD88-dependent pathway, since both IFN-β expression and IP-10/CXCL10 secretion are abrogated in MyD88 KO DCs (Figures 4B,D). TLR13, a PRR involved in sensing a specific sequence from bacterial 23S rRNA, is present in mice but not in humans (41, 42). It is possible that TLR13 may play a role in *B. abortus* RNA sensing, but this hypothesis needs further investigation. The assumptions aforementioned could also explain the results obtained in Figure 5, in which we compared bacterial clearance *in vivo* in WT, TLR3 KO, and TLR7 KO mice and we observed no significant differences among these studied groups. Similar findings were previously described by Oliveira and coworkers, where the macrophages derived from NOD1, NOD2, and RIP2 KO mice showed an altered IL-12 and TNF-α secretion but no differences were observed among these groups *in vivo* (43).

*Brucella abortus* has developed sophisticated molecular strategies to ensure its survival inside host cells by forming BCVs, which traffics from the endocytic vesicle to the ER, where the bacterium proliferates (44). BCV maturation is evidenced by the exclusion of LAMP-1, possibly by recycling LAMP-1^+^ membranes while interacting with the ER (45). Results shown in the Figure 3 demonstrate that, after 24 h of infection, *B. abortus* colocalized with TLR7 in BCVs that retain LAMP-1 labeling. However, it is important to highlight that literature on BCV maturation is based on studies using macrophages, and BCVs from infected DCs could present alterations on their membrane constitution. In addition, it is possible that in infected DCs ER-localized *Brucella* could hold membrane rearrangements dependent upon a subset of autophagy-associated proteins, producing vacuolar LAMP-1^+^ compartments that are phenotypically distinct from ER-derived BCVs, so called autophagic *Brucella*-containing vacuoles (44).

In summary, our findings shown here suggest that *B. abortus* RNA is an important immunostimulatory nucleic acid, and bacterial RNA-initiated TLR3 and TLR7 signaling contributes to cytokine response and type I IFN expression in murine DCs. To the best of our knowledge, this study reports for the first time the involvement of RNA sensing TLRs on innate immune responses against *Brucella* infection and highlights the importance of nucleic acid sensing during bacterial diseases.

## Author Contributions

PC, MG, and SO designed the project and experiments. PC, MG, EG, and GG carried out most of the experiments. PC and SO wrote the manuscript. PC carried out statistical analysis and prepared figures. SO submitted this paper. All the authors reviewed the manuscript.

## Conflict of Interest Statement

The authors declare that the research was conducted in the absence of any commercial or financial relationships that could be construed as a potential conflict of interest.
