# Supplementary material for: TLR7 and TLR3 Sense Brucella abortus RNA to Induce Proinflammatory Cytokine Production but They Are Dispensable for Host Control of Infection
Source: Front Immunol. 2017 Jan 23;8:28. doi: 10.3389/fimmu.2017.00028 (PMC5253617; doi:10.3389/fimmu.2017.00028)
Supplement: Supplementary file 1 [file Image_1.PDF]

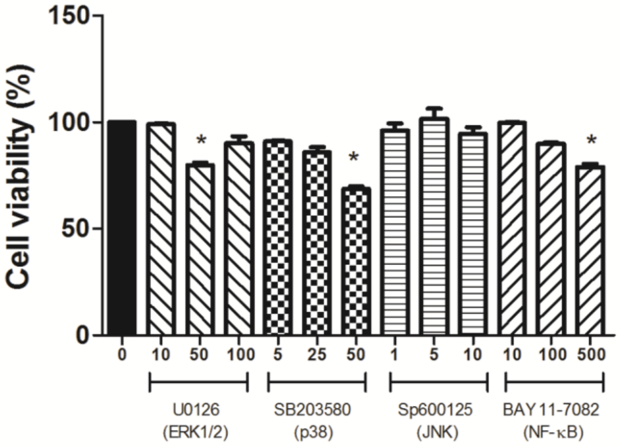

Supplementary Fig 1: MTS metabolism in bone marrow dendritic cells after incubation with different doses of MAPK/NF-κB inhibitors. DCs from C57BL/6 mice were kept in 96-well plates for 24 hours in 10% FBS-supplemented RPMI. Then the medium was replaced with 10% FBS-supplemented RPMI + increasing concentrations (in µM) of U0126, SB203580, Sp600125 and BAY11-7082 (ERK1/2, p38, JNK and NF-κB inhibitors, respectively) or vehicle (0.1% DMSO, black bar), as indicated. After 30 min of incubation at 37ºC 5% CO_2, cell viability was assayed using a MTS colorimetric assay (CellTiter 96 ® Aqueous One Solution kit, Promega, USA) according to manufacturer's instructions. Colorimetric reactions were measured with a spectrophotometer at 490 nm. Significant differences in relation to vehicle treatment are denoted by one asterisk (for $P<0.001$, one-way ANOVA test).
